# Parallel LC shaped metamaterial resonator for C and X band satellite applications with wider bandwidth

**DOI:** 10.1038/s41598-021-95468-8

**Published:** 2021-08-10

**Authors:** Mohammad Rashed Iqbal Faruque, Air Mohammad Siddiky, Eistiak Ahamed, Mohammad Tariqul Islam, Sabirin Abdullah

**Affiliations:** 1 Space Science Centre (ANGKASA), Institute of Climate Change (IPI), Universiti Kebangsaan Malaysia, Bangi, 43600 UKM Selangor Malaysia; 2 Department of Electrical, Electronic and Systems Engineering, Universiti Kebangsaan Malaysia, Bangi, 43600 UKM Selangor Malaysia

**Keywords:** Electrical and electronic engineering, Characterization and analytical techniques

## Abstract

The electromagnetic properties of the metal based dielectric in the field of millimeter and sub-millimeter technology attracts a new era for innovation. In this research work, we have introduced a parallel LC shaped metamaterial resonator with wider bandwidth. The negative refractive index for two resonant frequencies is located from the negative permittivity from 5.1 to 6.3, 10.4 to 12.9 GHz, where the negative refractive index is located from 5.4 to 6.3 and 10.5 to 13.5 GHz. The electromagnetic wave polarizing in the proposed structure with parallel LC shaped metallic structure shows a fascinating response of wider bandwidth for the external electric and magnetic field. This paper focuses on the design of conducting layer for the suggested design with the parallel metallic arm for analysing the mutual coupling effect of the scattering response where the sub-branch in metallic design is shown more resonant frequencies with the enhancement of the compactness. This proposed structure is analysed with different metallic arrangements and array structures for different boundary conditions.

## Introduction

The artificial inclusion of metamaterial based resonator in subwavelength range introduces exotic properties of electromagnetic waves. This artificial subwavelength structure can be engineered by geometric specification^1^. Improvement in engineering and technology, allowing macroscopic characteristics that present a broad range of opportunities for different fields of applications such as absorber, a different type of microstrip patch antenna, mechanical metamaterial, tunable metamaterial, sensing, cloaking, energy harvester, biomedical imaging, coding metamaterial, SAR reduction, filters, etc.^2–17^. The interesting feature of the metamaterial is that the polarisation characteristics of the metal-dielectric structure for the external electromagnetic waves interaction with the specific boundary conditions do not achieve in the normal way. Metamaterials show strong manipulation of the electric field and magnetic field by different orientations, the intensity of the applied source, polarisation angle, phase and geometrical structure. The metallic strips and split wire introduced inductive and capacitive effects resulting in resonant mode by governing the geometrical arrangement. The dielectric-metallic structure with the split wire possesses the strong electric field polarisation for the given EM wave that leads to single negative permeability (Mu-negative metamaterial) or single negative permittivity (epsilon-negative metamaterial) or both (double negative metamaterial) within a certain microwave frequency. The metamaterials are the next level of structural organization of the matter, which attracts scientists to improve the performance characteristics by different techniques and methods on the basis of propagating EM wave properties^18–20^.

The researchers in recent decades have extended their dedication to introducing the new expect of future technology to utilize the exciting properties of electromagnetic waves. A four-port MIMO(multi input multi output) antenna with mutual coupling was used for achieving circular polarisation. A metamaterial with slow wave characteristics is embedded in the proposed antenna to refine the electromagnetic characteristics^21^. A Vivaldi antenna in the THz spectrum with high gain and directivity was designed for medical sensing with reconfigurable characteristics. The graphene slabs are embedded in the antenna structure to improve the bandwidth which affects the gain and return loss of the antenna^22^. The enhancement of the gain and bandwidth with p-i-n diode switching a fractal ground loaded monopole antenna was designed for the enhancement of the impedance bandwidth^23^. Hexagonal nested loop double negative metamaterial-based MIMO antenna implemented to reduce the antenna element by absorbing the near field component of the magnetic field^24^. Minkowski-like fractal geometry was introduced with a composite right/left-handed transmission line for size miniaturization for the enhancement of the multiband performance that had omnidirectional radiation with a low cross-polarisation level^25^. The metamaterial with multi parallel rings embedded for the size miniaturization of the omnidirectional antenna showed a 60% frequency shift^26^. Various arrangements of the effective changing length by adding reconfigurable characteristics to the fractal microstrip antenna for the improvement of the bandwidth and higher gain using frequency selective surface (FSS)^27^. The fabricated substrates with nickel oxide nanoparticles deployed in polyethylene called INP substrate and metamaterial printed antenna with silver nanoparticles conductive ink used for microwave applications including RF energy harvesting^28^. Near-Zero-Index based square enclosed circular split ring resonator is analysed for S-,C- and X- band applications using Rogers substrate where the unit metamaterial unit cell is designed with two split ring resonators^29^. The return loss, power gain and efficiency improved using composite periodically layered metamaterial with non-magnetic metal cylinders of the circular cross section on the rectangular patch antenna^30^.

Researchers get involved in linking the emerging phenomena of electromagnetic properties of metamaterial for different fields of applications. The limitation of the metamaterial based characteristics for some applications is that narrow bandwidth. In this paper, we introduce a parallel LC shaped 13 × 10 mm^2^ metamaterial structure for C-, X band application with the higher bandwidth of scattering parameters from 4.2 to 5.0 and 8.9 to 11.8 GHz.

### Unit cell dimension specification

**Figure 1:**
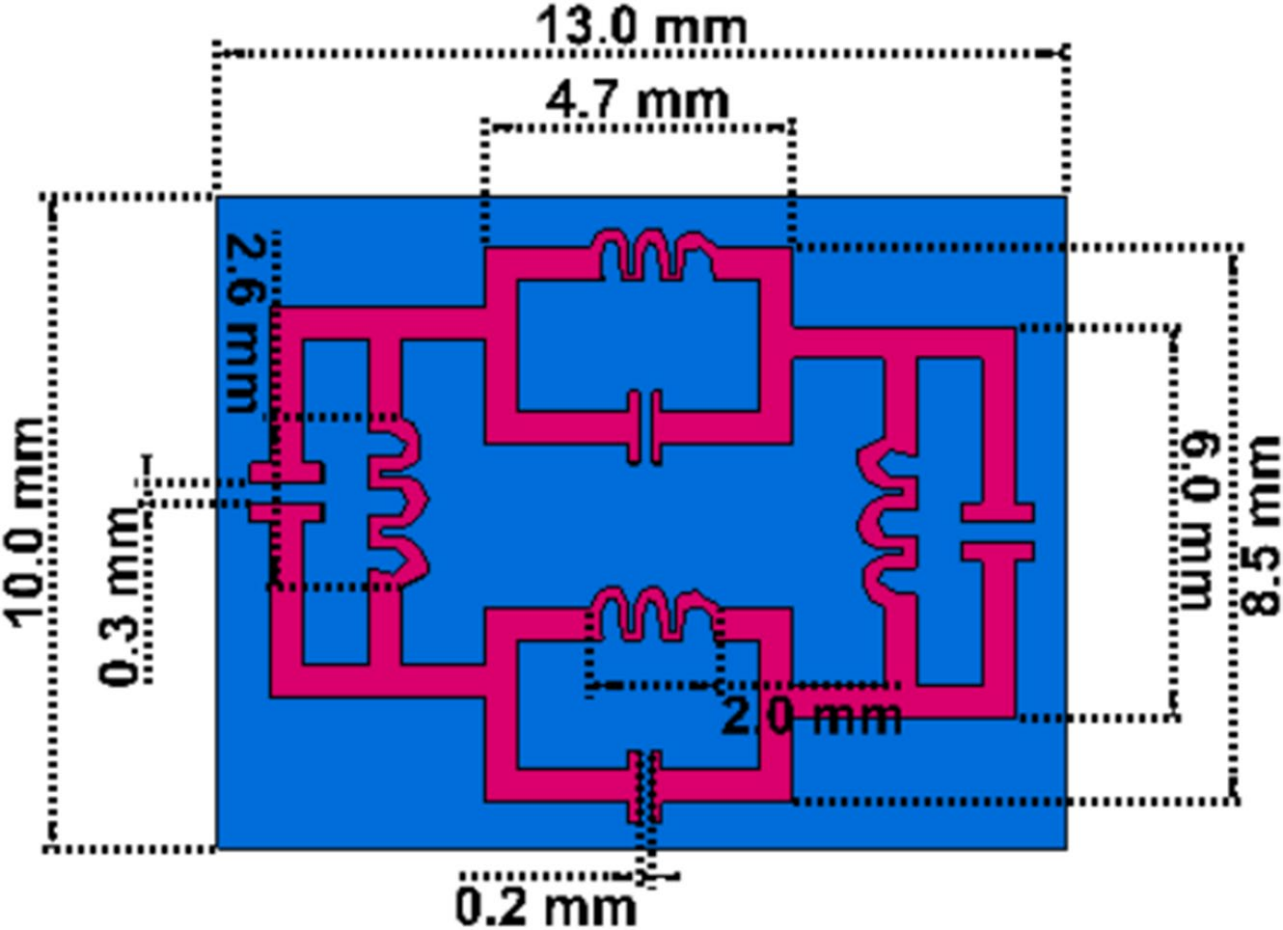
Geometric specification for suggested metamaterial.

**Figure 2:**
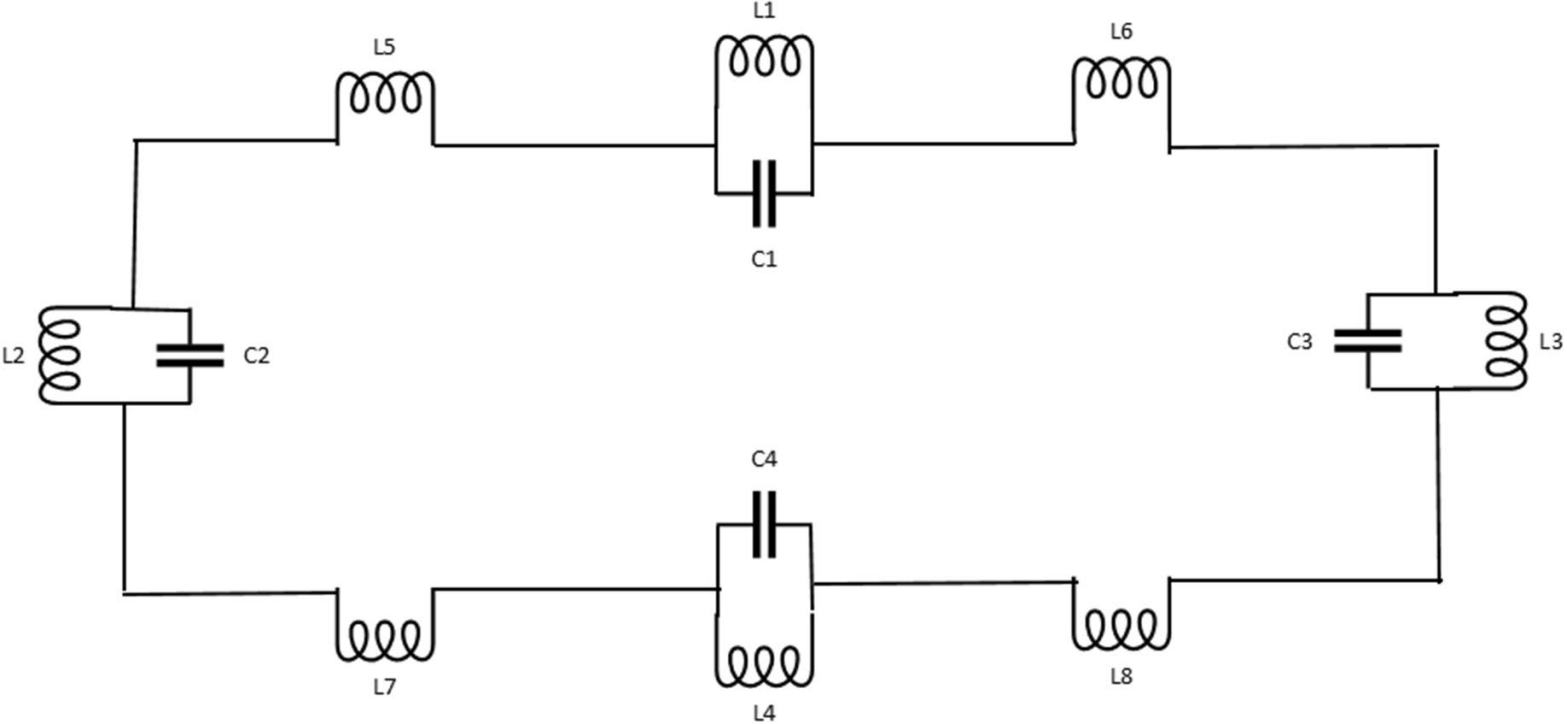
LC based equivalent circuit model for proposed metamaterial resonator.

**Figure 3:**
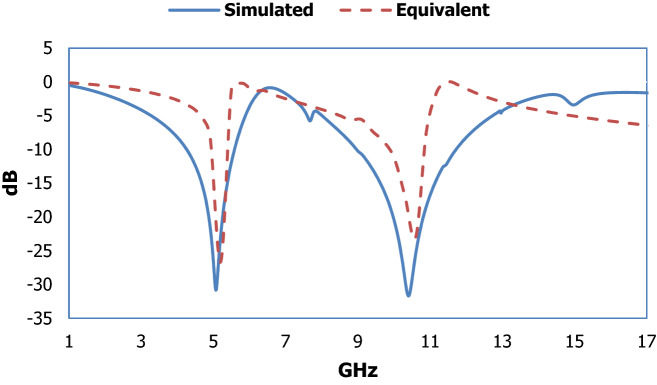
Scattering parameters extracted from high frequency simulator and equivalent circuit model.

The unit cell structure for metamaterial with different dimensional lengths is shown in Fig. 1 and the specification for the different diameters is given in Table 1. LC-shaped two parallel branches are placed on a vertical position with a dimension of 4.70 mm along the Y-axis, whereas the length of the spiral structure is 2 mm and the gap between the capacitive split is 0.2 mm. Another two parallel LC tank circuit is placed on horizontal position with the dimension 6.00 mm along X-axis, where the spiral structure is 2.6 mm and the capacitive split is 3.00 mm. This geometric specification depicts two types of metallic layer design strategy for the applied electromagnetic field where inductive shaped metallic arm exhibit the tunability within the operating frequency range and parallel branch exhibit the mutual coupling effect. The equivalent circuit model and the corresponding result are shown in Figs. 2 and 3 successively, which is validated by ADS software. The metallic structure with the split arm printed on a dielectric substrate working as LC network produces resonant frequency within an operating frequency range. The electric field is involved with the metallic gap that introduces capacitive effect and the metallic arm acts as an inductance that influences by the applied time-varying magnetic field. The negative electric susceptibility and negative magnetic susceptibility produced in the metal-dielectric interference in the subwavelength region. The Incident electric field vector is applied parallel to the split of the gap producing dip transmission spectrum and the external electric and magnetic field could couple with the magnetic SRR resonance. This artificial subwavelength inclusion is related to the periodic boundary condition of the defined electric and magnetic fields. In Fig. 3 shows that the equivalent circuit model for the proposed metamaterial resonator, where (L1, C1), (L3, C3) deployed for vertical parallel LC tank circuit and (L2, C2) and (L4, C4) deployed for horizontal LC tank circuit. Metallic arm used for joining parallel LC tank circuit represented as L5, L6, L7 and L8. The inductance and capacitance can be expressed^31^ as below,

1 $${\text{C}}_{{\text{e}}} = {\text{C}}(1 + {\varvec{\omega}}^{2} /\omega_{0}^{2} )$$

2 $${\text{L}} = 2 \times 10^{ - 4} l\left[ {ln\left\{ {\frac{2l}{d} + \sqrt {1 + \left( \frac{2l}{d} \right)^{2} } + \frac{d}{2l} - \sqrt {1 + \left( \frac{d}{2l} \right)^{2} } } \right\}} \right]$$

**Table 1:** Dimension specification for the metamaterial resonator.

Length of the Parallel LC arm vertically (mm)	Length of the parallel LC arm horizontally (mm)	Length of inductive turn (mm)	Gap for capacitve coupling vertically (mm)	Gap for capacitive coupling horizontally (mm)	The length of the substrate (mm)	The width of the substrate (mm)	Thickness of the metallic layer (mm)
4.7	6.0	2.0	0.2	0.3	13.0	10.0	0.035

The total capacitance and inductance for a single loop can be defined as:

3 $${\text{C}}\,({\text{pF}}) = \frac{{\varepsilon_{e} 10^{ - 3} \,K\left( k \right) }}{{K^{\prime}\left( k \right)}} (N - 1)\,l$$

4 $${\text{Where}},\,{\text{K}} = tan^{2} \left( {\frac{a\pi }{{4b}}} \right),\,a = \frac{w}{2},\,b = \frac{w + s}{2}$$

5 $$L_{n} (H) = 1.257 \times 10^{ - 3} \,{\text{a}}\left[ {\ln \left( {\frac{a}{w + t}} \right) + 0.078} \right]K_{g}$$

6 $${\text{Where}},\,{\text{a}} = \frac{{D_{0} + D_{i} }}{4},\,{\text{c}} = \frac{{D_{0} - D_{i} }}{2}.$$

### Design procedure

**Figure 4:**
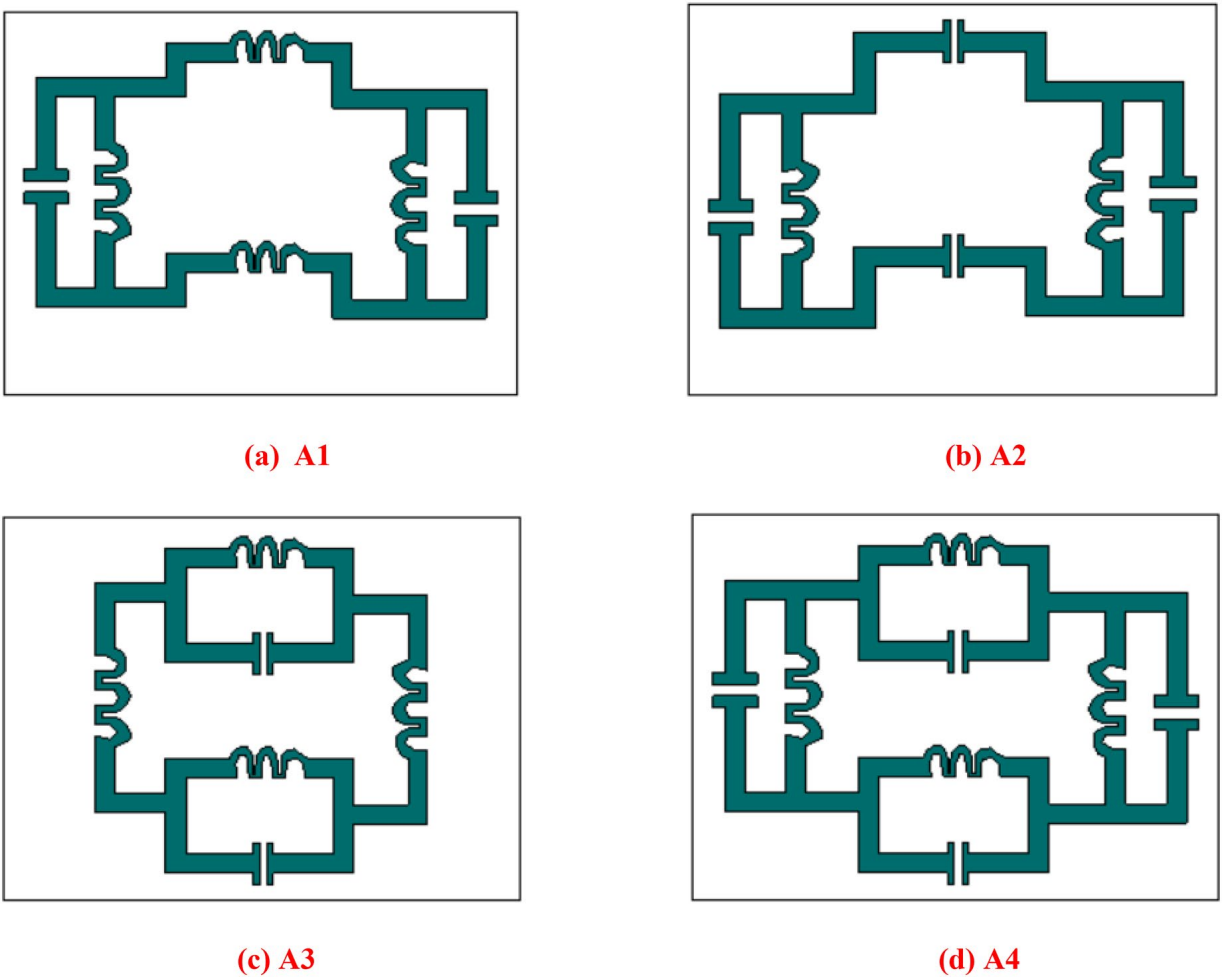
Different designs for the geometrical assessment.

**Figure 5:**
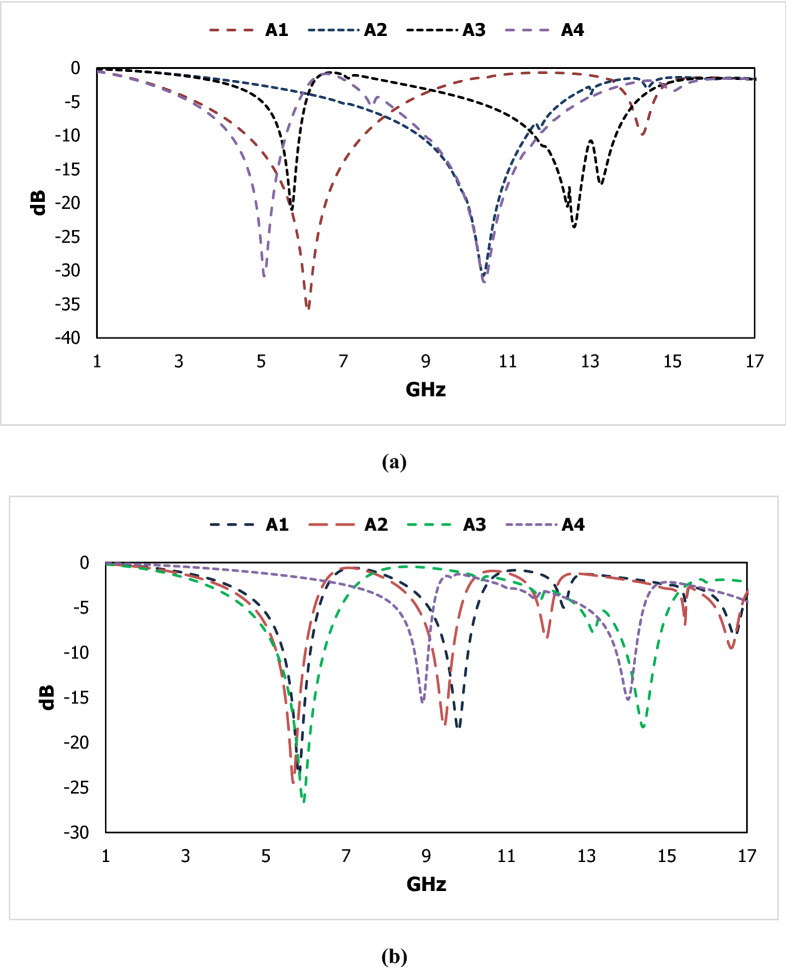
Results for two types of boundary condition with different geometrical structure.

Figure 4 shows that the different metallic structure arrangement to observe the characteristics for selecting the fabricated design and the transmission coefficient for different boundary conditions is given in Fig. 5. The transmission coefficient results are shown in Fig. 5a, where the electric field is given in X direction and the magnetic field in Y-direction. The magnetic field is given in the X-direction and the electric field in the Y direction for the results of the transmission coefficient in Fig. 5b. Two inductive shaped vertically and parallel LC shaped circuits horizontally represented in Fig. 4a, two series capacitive coupling with two parallel LC tank circuits introduced in Fig. 4b, two inductive couplings with two LC tank circuits vertically shown in Fig. 4c and the proposed structured with two LC tank circuit placed in the horizontal and vertical position is designed in Fig. 4d. For vertical magnetic field excitation and horizontal electric field excitation, design A1 produces one resonant frequency at 6 GHz; design A2 produces a single resonant frequency below 11 GHz; design A3 shows an abrupt response for the second resonant frequency and the proposed A4 design shows two resonant frequencies from 4.2- 5.0, 8.9- 11.8 GHz. The designs A1, A2, A3 and A4 show multiple resonant frequencies with narrow bandwidth within operating frequency range for the excitation of the magnetic field horizontally where the electric field is functioning perpendicularly.

## Methodology

**Figure 6:**
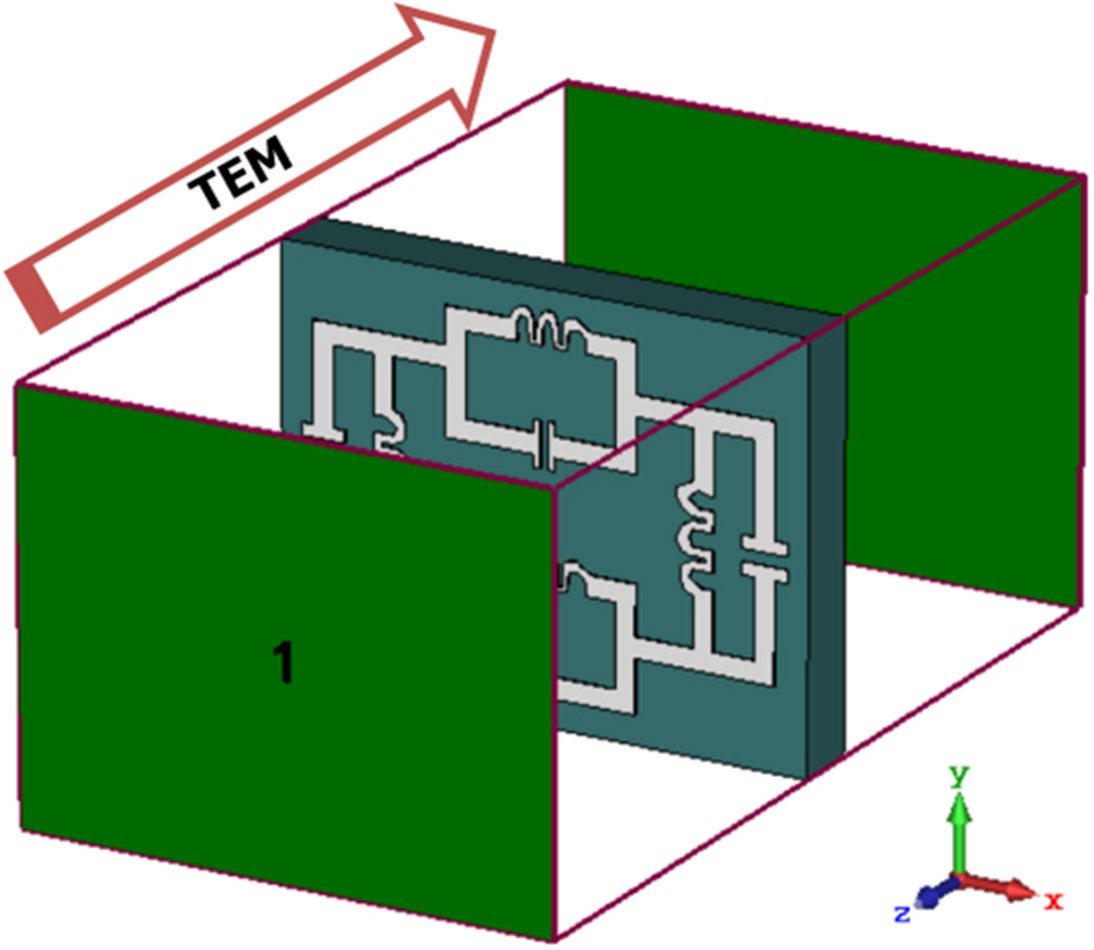
Boundary setup of the metamaterial cell for the propagated electromagnetic wave.

**Figure 7:**
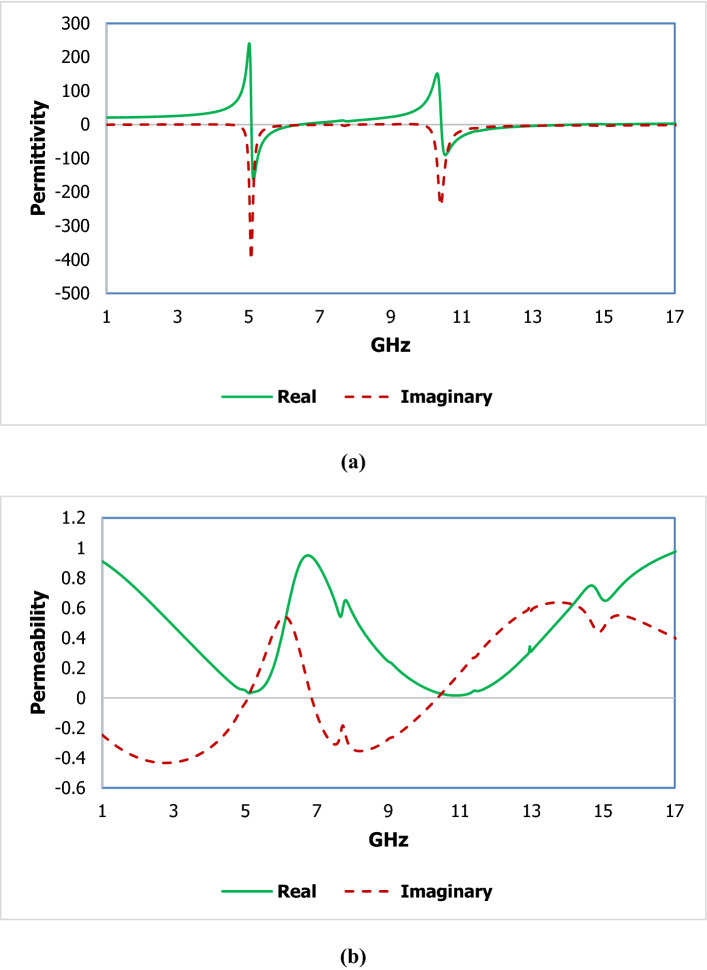
The effective parameters (**a**) permittivity and (**b**) permeability within the operating frequency range.

The electromagnetic properties for metal–insulator subwavelength structures are analysed from the scattering parameters through the numerical method and fabrication process. The simulated data of the transmission coefficient and reflection coefficient is calculated using a high-frequency microwave simulator CST microwave studio 2019 using the FEM technique. The boundary condition is provided to give the specific area of the model, impart the model to stabilize solver, suppress the irregularities of the specified model, proper interaction of the electromagnetic energy. The transverse electromagnetic wave is propagated to the proposed design in the Z direction which is shown in Fig. 6. The time-varying electric field is given in the X direction, where the time-varying is given in the Y direction. Polarisation due to the electric field and magnetic field takes place in the perpendicular direction and parallel with a split region of the subwavelength metallic structure. The finite metamaterial slab is used to determine the effective parameters of the subwavelength inclusion. The electric permittivity defines the polarisation effects of the dielectric medium with electric susceptibility, where permeability is related to the degree of magnetisation through the metallic strip with magnetic susceptibility and the refractive index is related to the electromagnetic propagation through the dielectric substrate. A magnetic dipole resonance is related to the circulating electric displacement current and electric resonance is related to the circulating magnetic field. A retrieval procedure^32^ is applied in the subwavelength material inclusion to measure the transmission and reflection coefficients by MATLAB software which is shown in Fig. 7 and the region of the effective parameters is shown in Table 2.

**Table 2:** The effective parameters extracted from MATLAB.

	GHz
Permittivity (real negative)	5.1–6.2. 10.4–13.1
Permeability (real near zero)	10.7–11.1

The average field flux and intensity are related to the equivalent electromagnetic parameters that determine the subwavelength structure's effective parameters.

7 $${V}_{1}= {S}_{21}+{S}_{11}$$

8 $${V}_{2}={S}_{21}-{S}_{11}$$

The scattering parameters of the proposed SRR design n be expressed as follows:

9 $${\text{Reflection}}\,{\text{coefficient}},\,S_{11} = \frac{{\left( {1 - {\Gamma }^{2} } \right){\mathcal{Z}}}}{{1 - {\Gamma }^{2} {\mathcal{Z}}^{2} }}$$

10 $${\text{Transmission}}\,{\text{coefficient}},\,S_{21} = \frac{{\left( {1 - {\mathcal{Z}}^{2} } \right){\Gamma }}}{{1 - {\Gamma }^{2} {\mathcal{Z}}^{2} }}.$$

The equation of the effective permittivity (${\varepsilon }_{r}$) is as follows:

11 $${\varepsilon }_{r}=\frac{2}{j\pi fd}\times \frac{(1-{S}_{21}-{S}_{11})}{(1+{S}_{21}+{S}_{11})}$$

The equation of the effective permeability (μ_r_) can be expressed as:

12 $${\mu }_{r}= \frac{2}{j\pi fd}\times \frac{(1-{S}_{21}+{S}_{11})}{(1+{S}_{21}-{S}_{11})}$$

13 $${\text{Where}}\;{\text{Z}} = \sqrt {\frac{{\left( {1 + S_{11} } \right)^{2} - S_{21}^{2} }}{{\left( {1 - S_{11} } \right)^{2} - S_{21}^{2} }}}$$

## Experimental measurement

**Figure 8:**
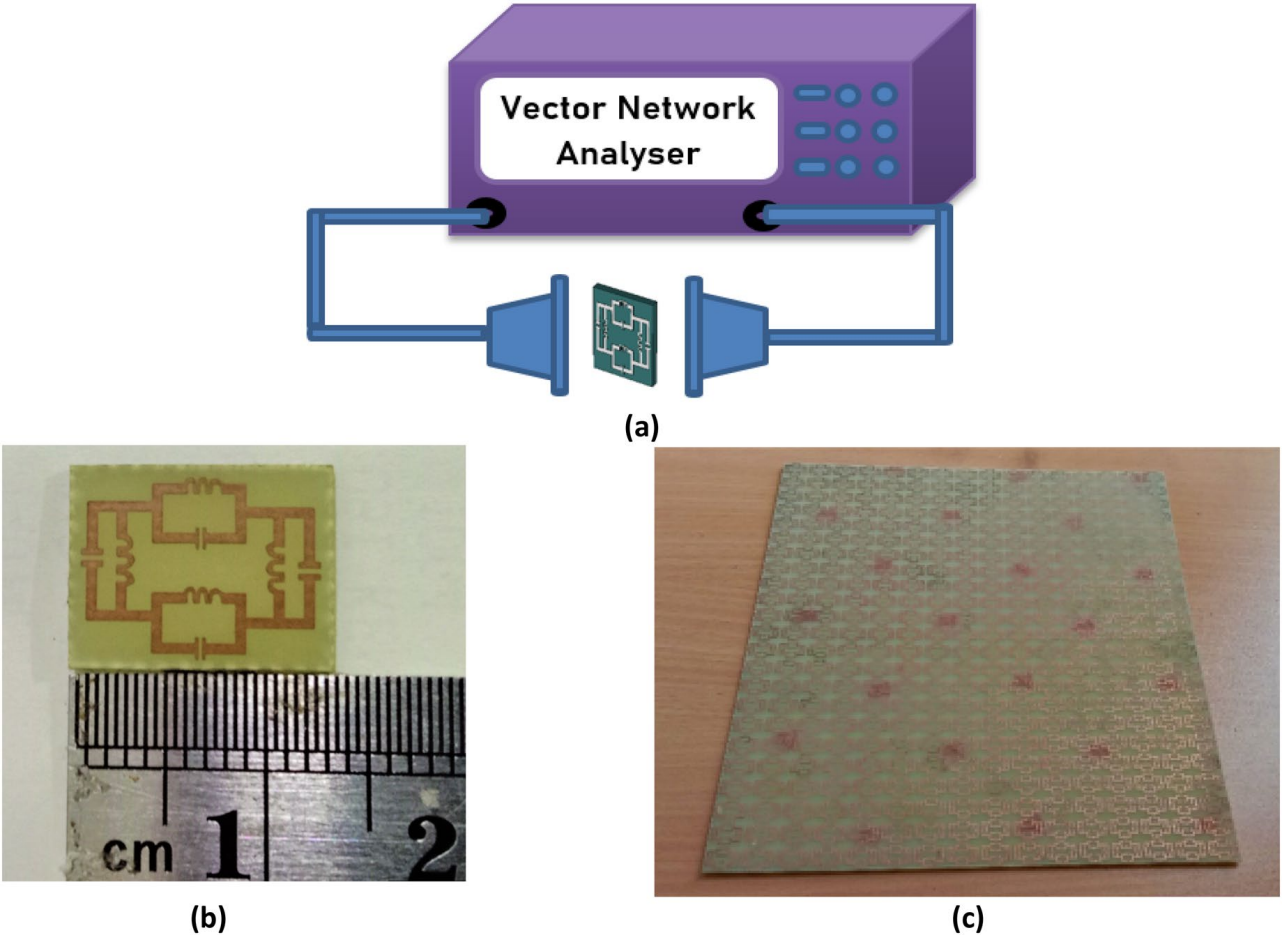
Vector network analyser with the peripheral adapter (**a**) and prototype of the unit cell (**b**) and array cell (**c**) for the experimental measurement.

**Figure 9:**
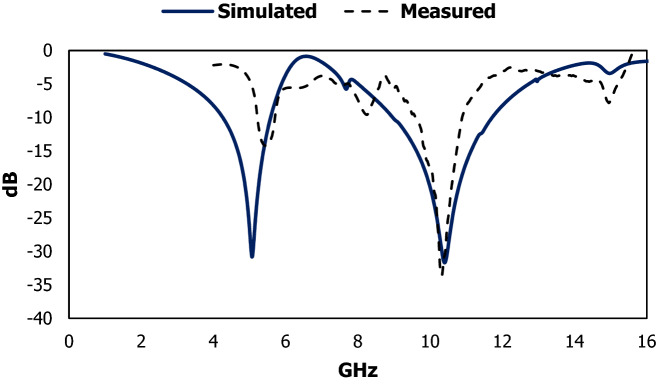
The simulated (blue line) and measured (black dotted) results for the LC shaped resonator.

The metallic sheet is deployed on FR-4 substrate used as dielectric medium and the proposed design is achieved through the etching process. The proposed metamaterial design is faced the waveguide port, where Y-axis directed the time-varying magnetic field, producing dipole in the X direction and the X-axis directed the time-varying electric field generating the dipole in the Y direction. To measure the experimental data for the suggested metamaterial of transmission coefficient applying time-varying transverse electromagnetic (TEM) field in the Z direction. Two waveguide port with peripheral cable is used for the source of TEM wave and connected with a vector network analyser to observe and extract the experimental value for scattering parameter which is shown in Fig. 8a. The prototype of the unit cell and array structure for the experimental measurement is shown in Fig. 8b,c. The array structure is placed between two antennas with a distance 3.7 m. The experimental data for the subwavelength inclusion exhibit irregularities at some points in Fig. 9 due to different factors such as signal leakage and reflection, EMI noise, switching loss, crystal defect, fabrication error, drift error, etc.

## Results and discussion

**Figure 10:**
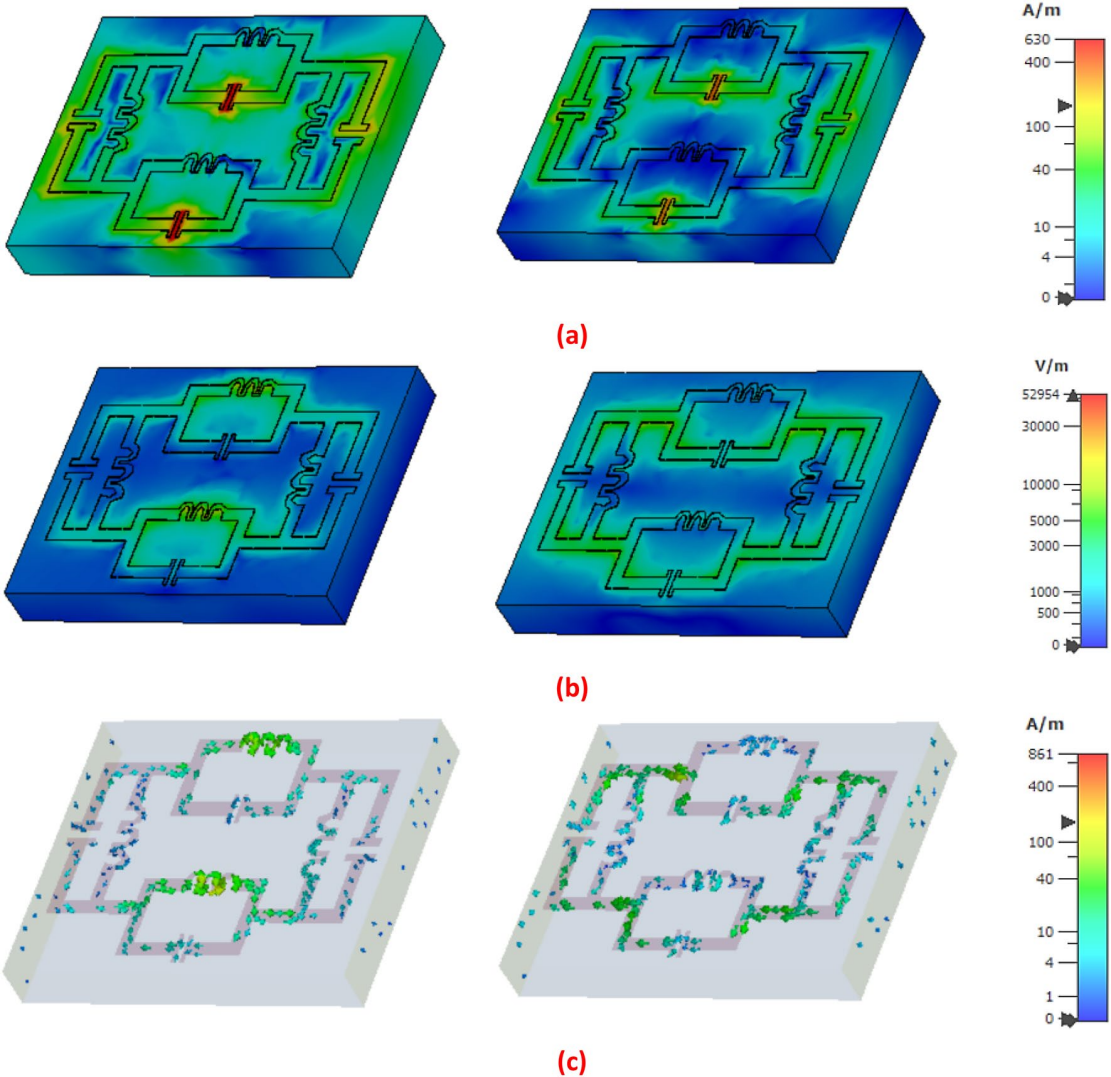
(**a**) Electric field distribution, (**b**) magnetic field distribution and (**c**) surface current distribution for the proposed parallel LC shaped resonator at 5.1 and 10 GHz.

The electric field distribution in fig shows strong accumulation in the capacitive coupling of the parallel LC branch along the horizontal direction, where the magnetic field firmly distributed on the inductive coupling along the vertical LC parallel circuit of the proposed design at 5.1 GHz. In Fig. 10, the electric field is distributed on the capacitive coupling of the four LC parallel circuit and intensely induced in the Y direction, where the magnetic field is distributed on the connector region of the four LC parallel LC-shaped metallic structure. The strong circulating current generating effective magnetic moment using subwavelength metallic structure lead to viable artificial magnetism. The surface current induced in the upper and lower inductive coupling of the LC parallel circuit in the clockwise direction and another pathway of the surface current follows the inward direction to the parallel LC branch and outward direction to the horizontal LC branch from the upward side lobe of the metamaterial structure at 5.1 GHz. The surface current on the metallic layer follows a clockwise direction through the capacitive coupled metallic arm and anticlockwise direction through the inductive coupled metallic arm of the parallel LC branch at 10.4 GHz.

### Parametric analysis

**Figure 11:**
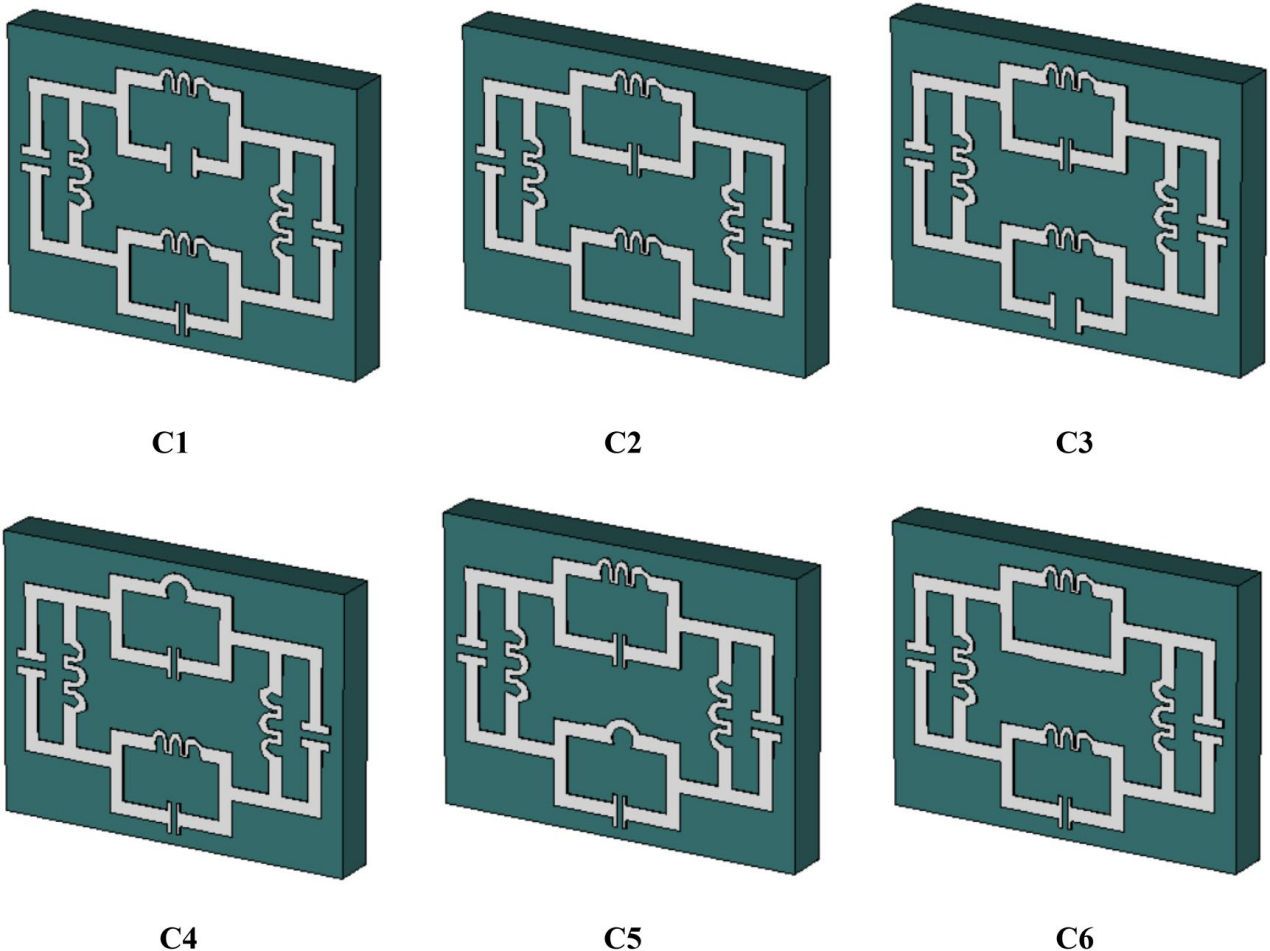
Different metallic designs for the observation of the inductive and capacitive coupling.

**Figure 12:**
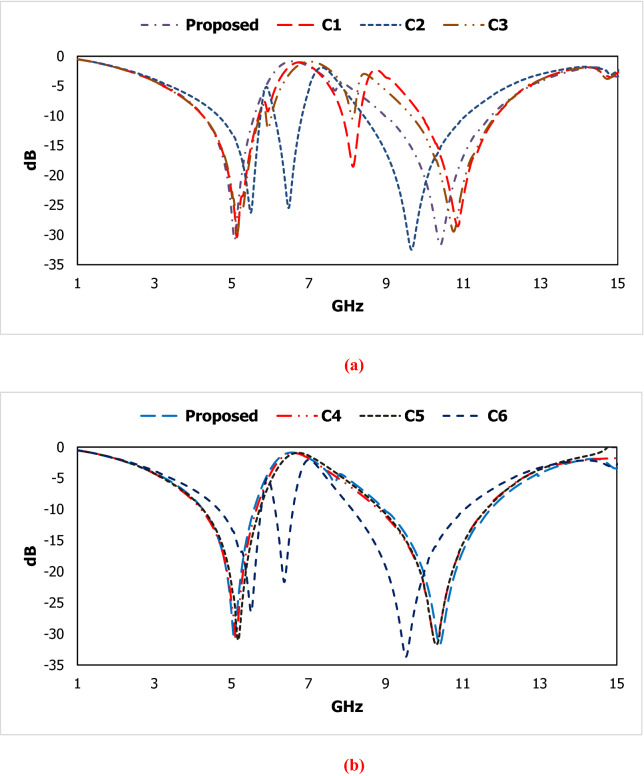
Results of scattering parameter for the different metallic designs.

The assessment for the parametric analysis by changing metallic structure is shown in Fig. 11 and corresponding results in Fig. 12. The variation in the geometrical arrangement in the metallic structure shows the effect of magnetic response related to metallic strip and electric response related to the split in a metallic arm of the metamaterial. The parametric data for different designs are shown in Table 3. As the increasing the split distance from 0.2 to 0.8 mm in the vertically positioned upper LC parallel branch of the metamaterial unit cell in design C1, introducing three resonant frequencies. The reduction of the bandwidth for resonant frequencies is observed within the operating frequency range. The capacitive coupling in the lower LC branch is short-circuited in design C2. This structure exhibit three resonant frequencies where two resonant frequencies are closer to each other and reduce the effective medium ratio. The irregular response of the scattering parameters for design C3 is shown in Fig. 12 due to the wider split distance in the lower LC branch. Reducing the inductive patch in the upper LC branch in design C4 exhibits a 100 MHz shift in the first resonant frequency and a 150 MHz shift is observed for design C5 by changing the inductive patch in the lower LC branch with referring to the proposed metamaterial structure. Using connected metallic structure in terms of capacitive coupling in design C6 is produced three resonant frequencies with narrow bandwidth. The proposed design exhibit a stable response to the above-mentioned designs.

**Table 3:** Data specification for the parametric analysis.

		C1	C2	C3	C4	C5	C6	Proposed
Capacitive gap in the parallel LC structure (mm)	Upper branch	0.8	0.2	0.2	0.2	0.2	_	0.2
	Lower branch	0.2	_	0.8	0.2	0.2	0.2	0.2
Capacitive coupling in the parallel LC branch	Upper branch	yes	yes	yes	yes	yes	no	yes
	Lower branch	yes	no	yes	yes	yes	yes	yes
Inductive turns in parallel LC structure	Upper branch	3	3	3	1	3	3	3
	Lower branch	3	3	3	3	1	3	3
resonant frequency		5.2, 8.3, 11.0	5.6, 6.4, 9.8	5.2, 8.3, 10.9	5.2, 10.4	5.3, 10.4	5.6, 6.5, 9.7	5.1, 10.4
Region of Bandwidth		4.33–5.76, 7.95–8.4, 10.0–12.1	4.72- 5.81, 6.2–6.9, 8.5–11.3	4.33–5.78, 8.29–8.31, 9.72–12.0	4.75–5.5, 8.9–11.7	4.8–5.6, 9.0–11.1	4.8–5.9, 6.2–6.9, 8.2–11.2	4.2–5.0, 8.9–11.8
Covered band		C-, X-, Ku	C-, X	C-, X-, Ku	C-, X	C-, X	C-, X	C-, X

**Figure 13:**
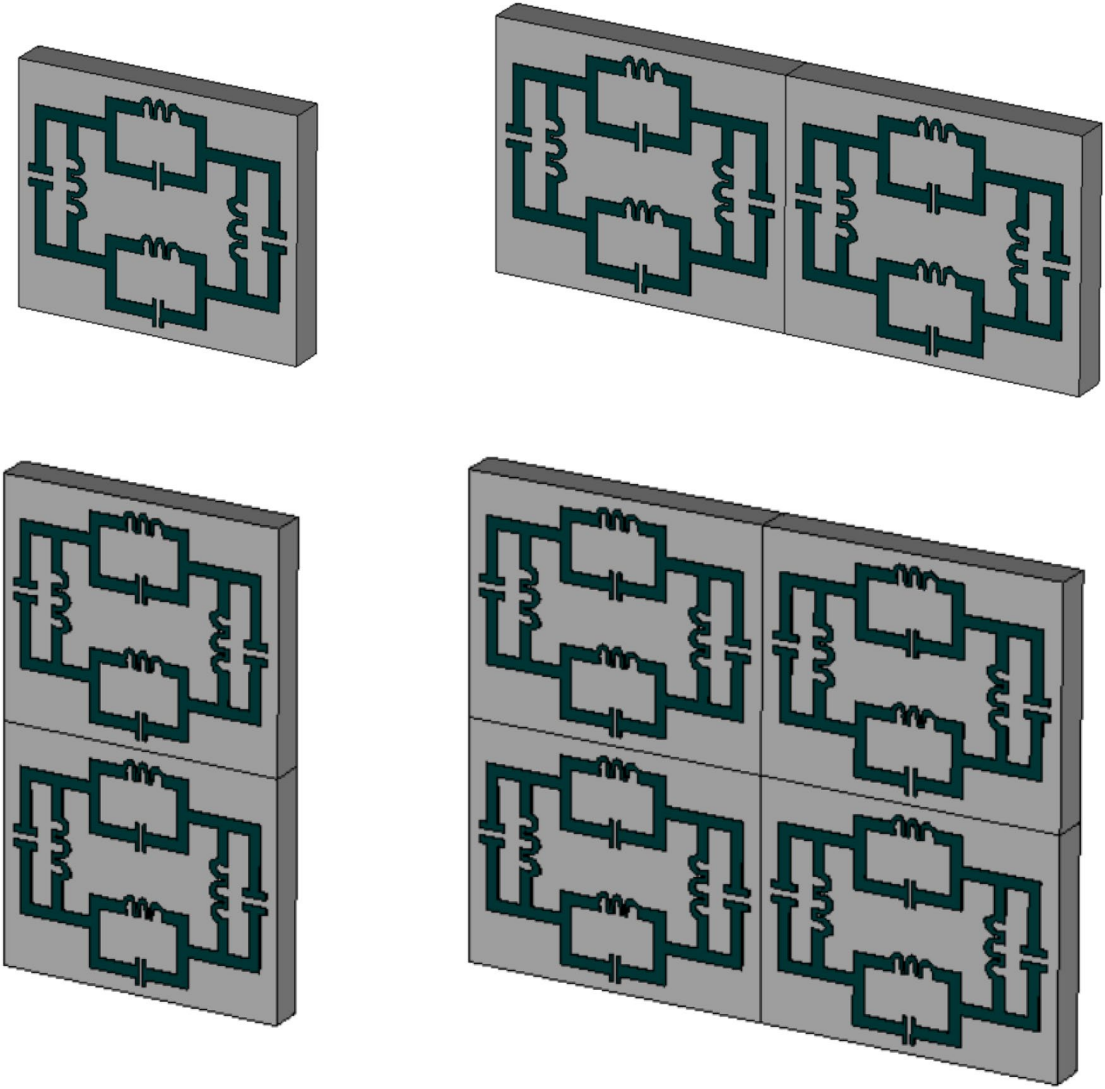
Geometrical arrangement for the unit cell, 1 × 2, 2 × 1 and 2 × 2 array cells.

**Figure 14:**
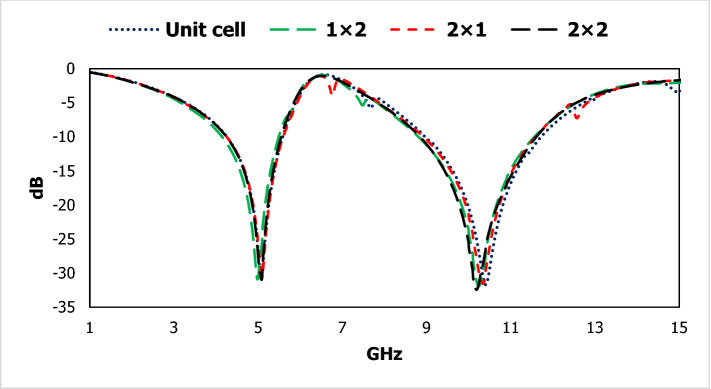
Results for different geometrical arrangements.

The exotic properties of the electromagnetic wave are observed with respect to the boundary condition interacted with the metal-dielectric-based subwavelength inclusion. Different type of array structure is observed for two types of boundary conditions in Figs. 13 and 14. The electric field is applied in the X-direction and the magnetic field in the Y direction for the first boundary condition, where the magnetic field is directed in X-axis and the electric field in the X direction for the second boundary condition. Different geometrical arrangements with the change of external field direction evaluate the effects of electromagnetic waves on the metamaterial slab. First boundary condition, we see that the 2 × 2 array cell is shown a smooth response than the other three types of structure. The unit cell, 1 × 2 and 2 × 1 array structures show unusual resonance frequency with lower gain amplitude from 6 to 8 GHz. The wider bandwidth provides the advantage of making the easy design for the integrated circuit.

The metamaterial design for different fields of applications attracts the researchers to analyse the exotic properties of the electromagnetic field with different shapes, substrate, dimension, number of resonant frequency, covered band, bandwidth, etc. Some recent papers are presented in Table 4 for the comparison with the established metamaterial design. A circular split ring resonator^33^ is printed on FR-4 substrate by Tayallen et al. with the bandwidth of 1000, 1200 GHz, Almutairi et al.^34^ with the bandwidth of 300, 900 GHz for dual resonant frequencies and T shaped metamaterial resonator^35^ is established with the bandwidth of 400, 900 GHz. Inverse double L shaped resonator designed on Rogers RT 5880^36^ with the bandwidth of 360, 410, 410, 360 GHz, square split ring resonator^37^ is established by Roy et al. using jeans as a substrate with the bandwidth of 300, 600, 1100 GHz and double H shaped resonator^38^ is used FR-4 substrate with the bandwidth of 410, 740, 1660 and 460 GHz. Circular cross-type metamaterial design layered on Rogers 2000^39^ for single-band applications with 150 GHz bandwidth and multi-split based resonator^40^ is designed for different band applications with the bandwidth of 100, 600, 700, 200, 500, 400 GHz. The suggested metamaterial design exhibit two resonant frequencies with the bandwidth of 800 and 2900 GHz. This feature of scattering parameters makes the design preferable for wideband faster communication applications.

**Table 4:** Comparison result with the different researchers established design.

	Shape	Substrate	Band	Resonant frequency	Band width	Year
^33^	Circular	FR-4	C-, Ku	2	1000, 1200	2019
^34^	Complementary SRR	FR-4	C	2	300, 900	2019
^35^	T shape	FR-4	C	2	400, 900	2018
^36^	Inverse double L	Rogers RT 5880	X-, Ku	4	360, 410, 410, 360	2019
^37^	CSRR	Jeans	S-, C	3	300,600, 1100	2019
^38^	Double H shape	FR-4	S-, X-, Ku	4	410, 740, 1660,460	2020
^39^	Circular cross type	Rogers 2000	S	1	150	2019
^40^	SSRR	Rogers RT 5880	S-, C-, X-, Ku	6	100,600,700, 200, 500, 400	2020
Proposed	Parallel LC type	FR-4	C-, X	2	800, 2900	2020

## Conclusion

A parallel LC-shaped resonator based metamaterial is designed for C-, X band applications with the optimistic results of scattering parameters. The wider bandwidth of transmission coefficient of this proposed metamaterial design makes it more durable and compatible with other wide range of microwave applications. High-frequency simulator CST 2019 is used to analyze the characteristics of the electromagnetic wave numerically and vector network analyser VNA N5227A is demonstrated for the assessment of experimental data. This metamaterial design exhibit the bandwidth from 4.2 to 5.0 and 8.9 to 11.8 GHz, where the metallic structure layered on FR-4 substrate. The wider bandwidth provides faster communication and easy signal encryption, wider filter, etc. The suggested design can be covered different satellite band applications such as satellite communication transmission, cordless phone, weather radar system, motion detectors and traffic light crossing detectors, etc.
